# Embedded Si/Graphene Composite Fabricated by Magnesium-Thermal Reduction as Anode Material for Lithium-Ion Batteries

**DOI:** 10.1186/s11671-017-2400-6

**Published:** 2017-12-16

**Authors:** Jiangliu Zhu, Yurong Ren, Bo Yang, Wenkai Chen, Jianning Ding

**Affiliations:** 1 School of Materials Science and Engineering, Changzhou University, Changzhou, 213000 China; 2 Jiangsu Province Cultivation Base for State Key Laboratory of Photovoltaic Science and Technology, Changzhou University, Changzhou, 213164 Jiangsu China; 3 College of Chemical and Chemical Engineering, Fuzhou University, Fuzhou, 350002 Fujian China; 4 Micro/Nano Science and Technology Center, Jiangsu University, Zhenjiang, 212013 China; 5 Department of Chemical and Biomolecular Engineering, University of California at Los Angeles, Los Angeles, CA 90095 USA

**Keywords:** Embedded Si/graphene composite, Magnesium-thermal reduction, Anode material

## Abstract

Embedded Si/graphene composite was fabricated by a novel method, which was in situ generated SiO_2_ particles on graphene sheets followed by magnesium-thermal reduction. The tetraethyl orthosilicate (TEOS) and flake graphite was used as original materials. On the one hand, the unique structure of as-obtained composite accommodated the large volume change to some extent. Simultaneously, it enhanced electronic conductivity during Li-ion insertion/extraction. The MR-Si/G composite is used as the anode material for lithium ion batteries, which shows high reversible capacity and ascendant cycling stability reach to 950 mAh·g^−1^ at a current density of 50 mA·g^−1^ after 60 cycles. These may be conducive to the further advancement of Si-based composite anode design.

## Background

Anode material plays a significant role in the rechargeable lithium-ion batteries (LIBs). Recently, most people think that the promising candidate for anode material are the silicon-based materials [1–3]. The main reason is that it has high theoretical capacity of 4200 mAh g^−1^ (about 10 times for the commercial graphite anode, 372 mAh g^−1^). In addition, there is abundant silicon in nature, and lithium insertion potential is relatively low (< 0.5 V vs. Li/Li^+^) [4–6].Unfortunately, there are limits to the commercialization of silicon-based anode materials. The reason why is that volumetric expansion of the Si electrode by over 400% can cause a series of problems such as electrode pulverization, poor cycling stability, and seriously irreversible capacity recession [7, 8]. Therefore, to solve the issue of volume expansion, lots of means have been proposed that include obtaining the nano-scale silicon particles, and preparing the silicon-based composites [9, 10]. For composites, the most efficient methods is dispersing the nano-scale silicon into the carbon matrix, where the carbon matrix functioned as buffer system and electroactive material [11]. Xuejiao Feng et al. prepared nano/μ-structured Si/CNT particles via a combination of spray drying and magnesium-thermal reduction with use nano-particles SiO_2_ as both a template and silicon precursor [12]. It exhibited a capacity larger than 2100 mAh g^−1^ at current density 1 A g^−1^, and the capacity retention after 100 cycles was 95.5%.

Recently, graphene, an original kind of carbon material, has aroused great concerns in the field of materials science. It has a unique structure with a single-layer sheet-like structure composed of carbon atoms [13]. Demonstrably, it is very promising to prepare some graphene-based materials with remarkable property due to the superior electrical conductivity and high surface [14]. Huachao Tao et al. designed a self-supporting Si/ RGO nano-composite films. The result indicated that the composite had admirable electrochemical performance [15].

In our work, we designed a novel method to synthesize high-capacity magnesium-thermal reduced Si/graphene (MR-Si/G) composite, which used the tetraethyl orthosilicate (TEOS) and graphene oxide (GO) as the starting materials, and was in situ generated SiO_2_ particles on graphene sheets followed by magnesium thermal reduction. Compared with the previous preparation method, the synthesis of materials in this experiment is relatively simple. At the same time, silicon and graphene are relatively evenly mixed by in-situ generated SiO_2_ particles on graphene. Embedded structure of the composite accommodated the large volume change, displayed high specific capacity and cycle stability, and increased the electronic conductivity. Another, the raw materials are cheap. All of these may be conducive to the further advancement of Si-based composite anode design.

## Experimental

Graphite oxide (GO) was obtained from flake graphite according to the modified Hummers method in literature [16]. Dispersing graphite oxide in deionized water to obtain 1 mg/ml aqueous solution. Then, take 30 ml anhydrous ethanol and 0.17 g cetyltrimethyl ammonium bromide (CTAB) blended by sonication for 10 min, then add 30 ml above graphite oxide aqueous solution and vigorous stirring to obtained mixture, followed by adding a specific amount tetraethoxysilane (TEOS) and magnetic stirring 10 min, finally the ammonium hydroxide was used to adjust PH to 10, then continually stirring 2 h. Lastly, the mixture was sealed with the Teflon-lined autoclaves at 180 °C for 10 h. The resulting compound was suction filtration and dried in vacuum at 60 °C for 24 h.

This step is to prepare the Si/G complex by magnesium thermal reduction. Firstly, the above composite was heated at 550 °C for 3 h at 5 °C/min in an argon atmosphere, and then cool it to room temperature. The weight ratio of as-sample and magnesium powder was 1:1 in an agate mortar and grinding 30 min. Then, the mixture was placed in a tube furnace and heated at 800 °C for 4 h in an argon atmosphere. Finally, the as-composite was soaked by 1 M HCl for 10 h then filtered and dried under vacuum at 60 °C for 8 h. This product is MR-Si/G composite.

The X-ray diffraction (XRD, D/max 2500PC) was used to characterize the phase composition of the materials. The morphology and structure of the products were evaluated by field emission scanning electron microscopy (FESEM, SUPRA55), Transmission electron microscopy (TEM, JEM-2100). Raman spectra and the FTIR Spectra were measured on RM2000 Raman Spectrometer (Renishaw, British) and the NICOLET 560 Fourier transform infrared spectrophotometer, respectively. The content of Si in the composite was measured by thermogravimetric analysis (TGA, NETZSCH TG 209F1 Libra), it was from room temperature to 800 °C at a heating rate of 10 °C/min under air.

To test the electrochemical performance, which was carried out in two-electrode 2032 coin-type cells, the active material (MR-Si/graphene), conductive additive (Super-P), and sodium carboxymethyl cellulose (CMC) as binder were intermingled at weight ratio of 80:10:10, which was used as the working electrode. The mixture slurry was prepared by using deionized water as a solvent, then equably pasted on pure copper foil current collector via doctor blade processing, followed by drying under vacuum at 105 °C for 12 h. All the cells were assembled in an argon-filled glovebox (ZKX2, Nanjing University Instrument Factory). The metallic lithium foil was used as the counter electrode. The electrolyte was a solution of 1.0 M LiPF6, which dispersed in mixture of EC: DMC: EMC (1:1:1 by volume). The cells were tested in the potential range from 0.01 V to 3 V (vs. Li+/Li) by CT2001A Land battery testing system.

## Results and Discussion

**Fig. 1:**
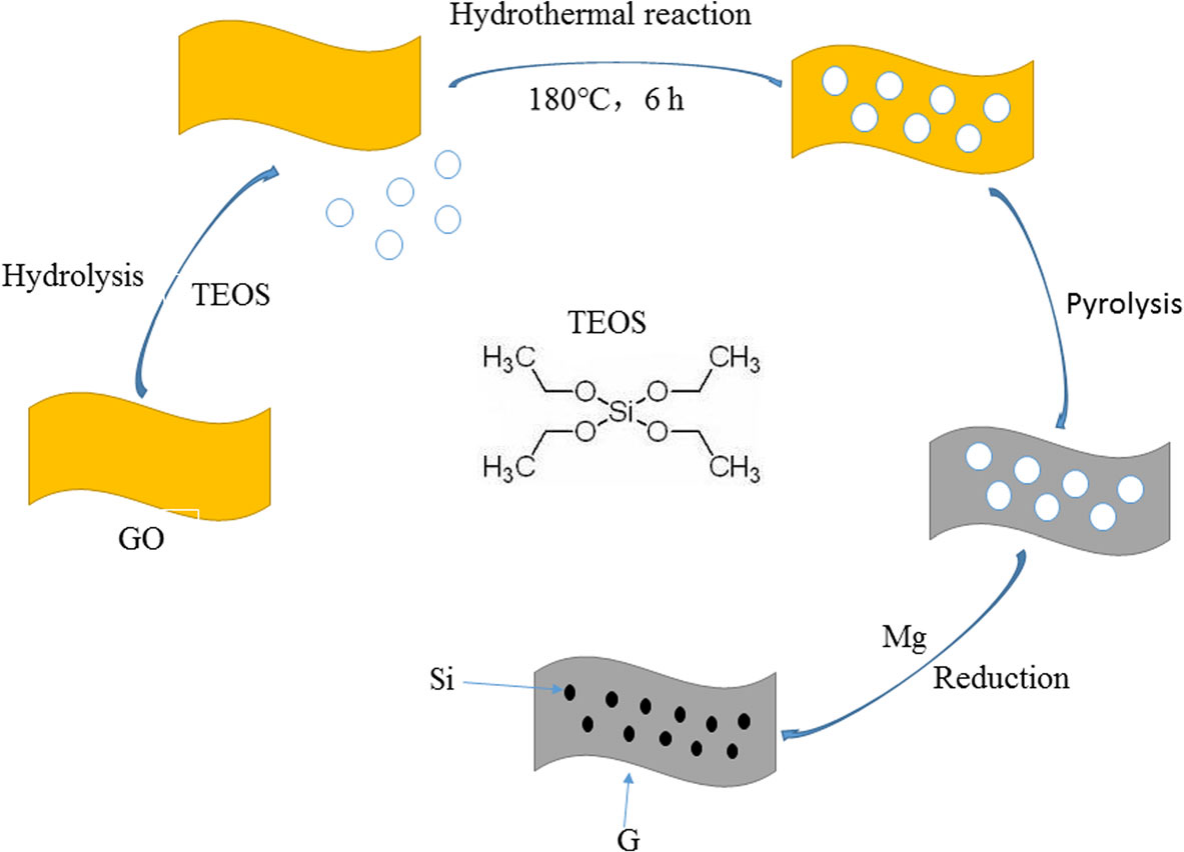
Schematic diagram of the preparation procedures for MR-Si/G

The MR-Si/graphene composite manufactured by in-situ generated SiO_2_ particles on graphene sheets followed by magnesium thermal reduction. Figure 1 illustrates the schematic diagram of the manufacture MR-Si/G complex. The SiO_2_ nano-particles were synthesized by modified Stöber process [17]. Subsequently, hydrothermal method was used to in-situ generate the SiO_2_/graphite oxide, the final composite was synthesized by magnesium-thermal reduction.

**Fig. 2:**
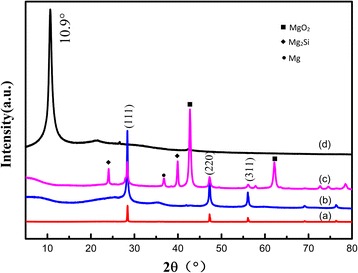
XRD profiles of graphite oxide, pure silicon, MR-Si/G composite

Figure 2 reveals the XRD pattern of Si, MR-Si/G and GO corresponding to (a), (b), and (d), respectively. Figure 2c is a composite material that has not been acid-treated. The reflection peak at 2ϴ = 10.9° is graphite oxide. The main diffraction peaks at 2ϴ =28.5°, 47.6° and 56.5° corresponding to the planes of (111), (220), and (311) typical of Si, which are distinctly observed in MR-Si/G compound and pure silicon. Compared the pure Si to the MR-Si/G composite in the XRD pattern, which indicated that add the graphite oxide without changing the structure of the compounds. However, the peak of graphite oxide in composite disappears, the reason why it may be restored into the graphene. In addition, the magnesium-thermal reduction is a key factor to successfully synthesize the novel compounds. Simultaneously, if Mg is excessive, there will be some side reaction. The reactions are as follows:

1 $$ 2\mathrm{Mg}+\mathrm{Si}\mathrm{O}2\to 2\mathrm{Mg}\mathrm{O}+\mathrm{Si} $$

2 $$ 4\mathrm{Mg}+\mathrm{SiO}2\to 2\mathrm{MgO}+\mathrm{Mg}2\mathrm{Si} $$

Compared Fig. 2b–c, magnesium and other by-products are removed by acid treatment.

**Fig. 3:**
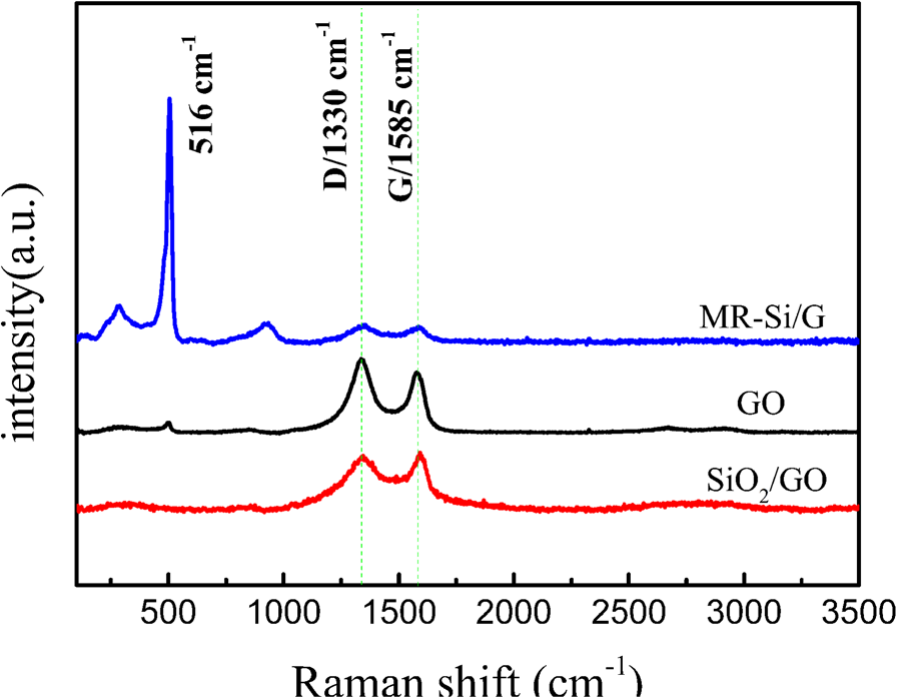
Raman spectra for graphite oxide, SiO_2_/GO and MR-Si/G composite

**Fig. 4:**
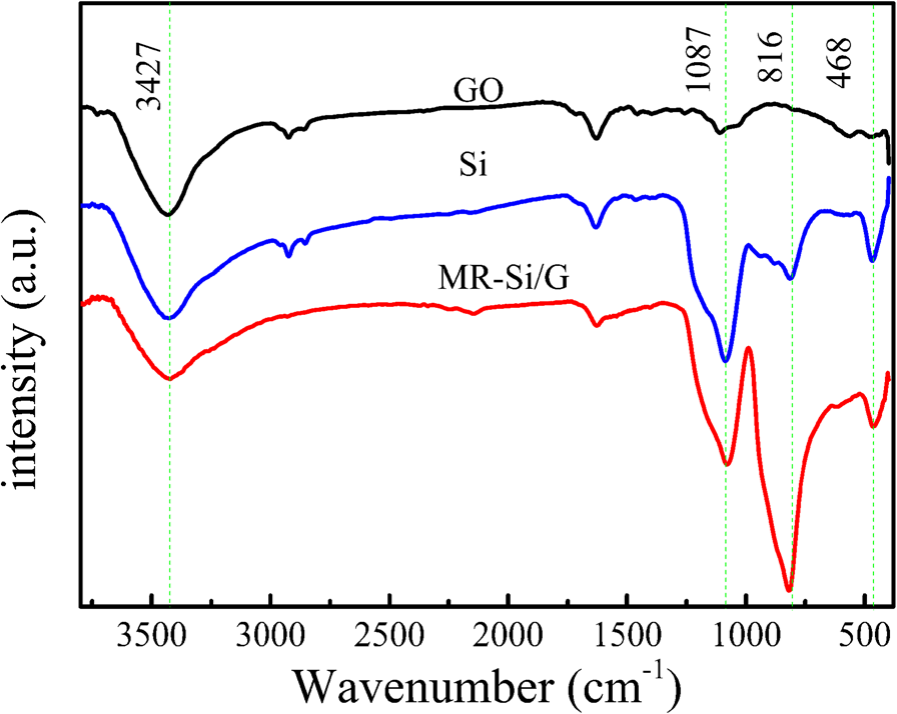
FITR spectra of the GO, pure Si, and MR-Si/G composite

From the Raman diagram in Fig. 3, MR-Si/G composite, the peaks at approximately 516 cm^−1^(this peak is absent in the SiO_2_/GO) is in accordance with the spectrum of Si nano-particle [18], manifesting that the silicon appeared after the magnesium thermal reduction. This result is consistent with the XRD. All the three curves, which the peaks at 1330 cm^−1^ and 1585 cm^−1^ consistent with the D band and the G band, respectively. The G-peak is the feature of the graphite, representing the carbon of the sp2 structure. The D-peak can be ascribed to the existence of a defective hexagonal graphite structure. The I_D_/I_G_ is the most important parameter, which was related to the graphitization degree of the carbonaceous material and the defect density in the graphene-based material [19]. Although it has been reported that the degree of ordering of graphene after thermal reduction is increased, the I_D_/I_G_ intensity ratios of MR-Si/G composite has increased, which maybe the presence of Si nanoparticles that increases the disorder of the material [20]. After calculation, the I_D_/I_G_ ratio of GO is approximately 0.93 and the I_D_ / I_G_ ratio of MR-Si / G is about 1.19. In order to further study the changes in chemical structure, we conducted FTIR to analyze the functional groups of sample. Figure 4 shows the FITR spectra of GO, pure Si, and MR-Si/G composite. For the Si and MR-Si/G composite, the peaks at about 468 cm^−1^, 816 cm^−1^, and 1087 cm^−1^ are corresponding to the O-Si-O bending vibration, symmetric elastic vibration of Si-O-Si and Si-O-Si asymmetric elastic vibration, respectively. The presence of these functional groups is conducive to the formation of a stable structure. And the broad peaks at 3427 cm^−1^ are related to the O-H stretching vibration.

**Fig. 5:**
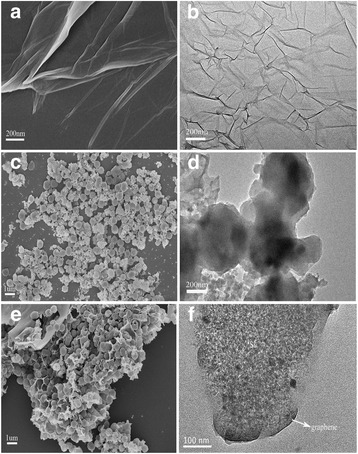
**a**, **c**, **e** shows the SEM images of the graphene, pure Silicon, and MR-Si/G composite, respectively. **b**, **d**, **f** is the corresponding TEM images, respectively

The morphology of all prepared materials was studied by SEM and TEM (Fig. 5). Figure 5a, c, e show the SEM images of the graphene, pure Silicon, and MR-Si/G composite, respectively. And the corresponding TEM images is Fig. 5b, d, f, respectively. We could see the morphology of graphene has many pleats and wrinkles, and the surface is relatively flat and smooth (Fig. 5a). TEM results are also matched (Fig. 5b). The nano-scale silicon particles are clearly seen to be spherical and dispersed evenly, but there is a ball-crushing phenomenon (Fig. 5c). The Si nano-particle size is about 500 nm in diameter. In the FE-SEM (Fig. 5e) and TEM images (Fig. 5f) of MR-Si/G composite, Si nano-particles distributed uniformly on the graphene and they are well embedded into graphene sheets. Comparing Fig. 5d with f, we can see that graphene layers exist at the edges of the composites.

**Fig. 6:**
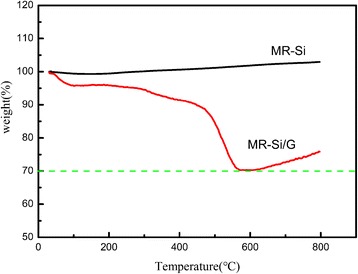
TGA curves of MR-Si/G composite and pure Si

The content of the Si in the MR-Si/G composite carried by the TGA measurements, which was implemented from ambient temperature to 800 °C with a heating rate of 10 °C/min in the air. As shown in Fig. 6, the start reaction temperature of the composite material is about 450 °C, and the oxidation reaction of the graphene oxide is completed at 600 °C. The weight loss of the composite represents the content of graphene, that is to say, the content of silicon in the complex also can be determined. From the picture, the weight percentages of Si is calculated to be about 70%. And in the complex, the curve increased above 600 °C, mainly due to the reaction of silicon with oxygen in the air to produce silica.

**Fig. 7:**
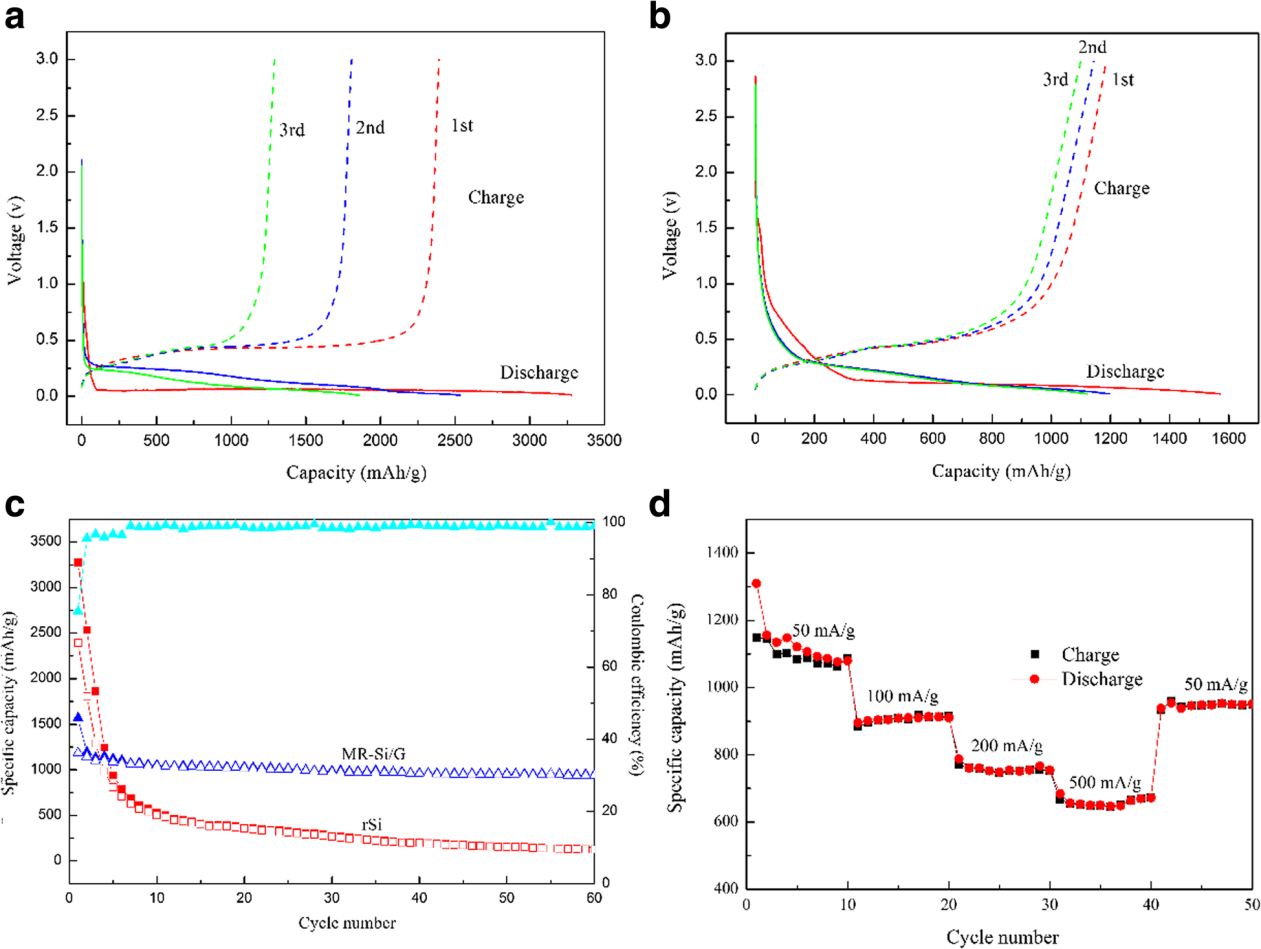
(**a**) The third charge and discharge curves of pure Si (**b**) The third charge and discharge curves of MR-Si/G composite (**c**) Cycling performance of MR-Si/G composite compared with pure Si (**d**) Cycling performance of MR-Si/G composite at various rates

Figure 7a, b depicts the first three times discharge-charge profiles of the pure Si and the MR-Si/G composite electrode, respectively. The current density is 50 mA·g^−1^ and voltage range of 0.01–3.0 V vs Li/Li^+^. For the pure Si, the initial discharge capacity is 3279 mAh·g^−1^, while the first charge capacity is only 2391 mAh·g^−1^(Fig. 7a).

For the MR-Si/G composite, the initial discharge capacity and charge capacity is 1570 and 1178 mAh·g^−1^, respectively (Fig. 7)b, and revealing coulombic efficiency of 75.5%. The large irreversible capacity may be put down to a solid electrolyte interface (SEI) film is formed on the electrode surface. The initial discharge curve demonstrates a long discharge flat curve with a plateau below 0.15 V. It can be attributed to the delithiation process from amorphous Li_x_Si phase [21]. As the number of cycles increase, the capacity continued to decline, but the decay rate is slower relative to pure silicon.

Figure 7c shows the cycle performance and the coulombic efficiency of the MR-Si/G composite compared to the pure Si at a current density of 50 mA·g^−1^ after 60 cycles. For the pure Si, the cycle performance is highly bad at the first 10 cycles, which the discharge capacity quickly dropped from 3279 to 528 mAh·g^−1^. After 60 cycles, the capacity was reduced to about 125 mAh·g^−1^. At the same times, the MR-Si/G compound has superior cycling properties, which the discharge capacity is 1570 mAh·g^−1^ and the reversible capacity is about 1055 mAh·g^−1^ in the initial 10 cycles. And the coulombic efficiency is achieved 99% and kept steady in a subsequent loop. It is noted that the specific capacity of complexes has been maintained at about 950 mAh·g^−1^ after 60 cycles. The results indicate that the graphene layers act as a significant role in the cycling performance of the compound electrode, which due to stabilizes the structure of the electrode and increases the electric conductivity. The rate capability of the MR-Si/G composite at different current densities is displayed in Fig. 6d. It is noted that the specific capacity of 1087,915,753 and 671 mAh·g^−1^ correspond to at the current densities of 50, 100, 200, 500 mA·g^−1^, respectively. In an addition, the capacity value is only 950 mAh·g^−1^ as the current density back to 50 mA·g^−1^.

**Fig. 8:**
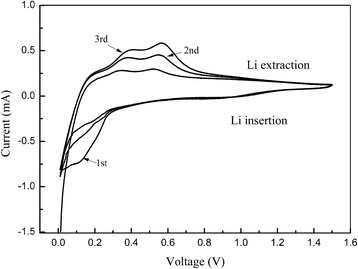
Cyclic voltammetry of MR-Si-G composite from 0.01 V to 1.5 V at a scan rate of 0. 1 mV s^−1^

Figure 8 shows the cyclic voltammetry of MR-Si-G composite from 0.01 V to 1.5 V at a scan rate of 0. 1 mV s^−1^. In the first cycle, the peak at 0.75 V during the cathodic sweep relates with the formation of SEI layer, which disappears in the next cycle. It matches the composite discharge curve (Fig. 7b). The peak at 0.16 V are related with the alloying reaction of Si and Li during the lithiation. Two anodic peaks at 0.31 and 0.50 V were observed during the delithiation, which could be attributed to the reaction between amorphous LixSi and amorphous silicon.

**Fig. 9:**
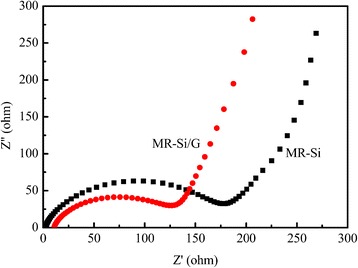
Electrochemical impedance spectroscopy (EIS) of the MR-Si/G and the pure Si

Figure 9 shows the electrochemical impedance spectroscopy (EIS) of the MR-Si/G and the pure Si. The downwardly diverging semicircle appearing in the high frequency region is related to the SEI impedance layer, and the oblique lines appearing in the low frequency region are related to the diffusion process of the lithium ions in the composite. In the figure, the impedance of MR-Si/G is lower than that of pure Si, indicating that the graphene significantly improves the conductivity of composite. The reason why is that not only the graphene has good conductivity, but also it can inhibit the cycle of SEI membrane changes, so as to promote the transfer of charge in the battery.

## Conclusions

Embedded Si/graphene nano-composite was successfully synthesized via combined with the hydrothermal process and Mg-assisted reduction. The Si nano-particles were fabricated by the magnesium-thermal reduction of amorphous silica nanoparticle, which were uniformly adhered on the graphene. The unique structure of the composite eases the volume expansion and manifests excellent electrochemical properties. The MR-Si/G composites exhibited high reversible capacity, which can be up to 950 mAh·g^−1^ at a current density of 50 mA·g^−1^ after 60 cycles. The methodology employed in this study produced a promising unique MR-Si/G composite, which for the next generation of high-capacity lithium-ion battery anode material, provides a reliable basis.
